# Spike Sorting by Joint Probabilistic Modeling of Neural Spike Trains and Waveforms

**DOI:** 10.1155/2014/643059

**Published:** 2014-04-16

**Authors:** Brett A. Matthews, Mark A. Clements

**Affiliations:** School of Electrical and Computer Engineering, Georgia Institute of Technology, Atlanta, GA 30332, USA

## Abstract

This paper details a novel probabilistic method for automatic neural spike sorting which uses stochastic point process models of neural spike trains and parameterized action potential waveforms. A novel likelihood model for observed firing times as the aggregation of hidden neural spike trains is derived, as well as an iterative procedure for clustering the data and finding the parameters that maximize the likelihood. The method is executed and evaluated on both a fully labeled semiartificial dataset and a partially labeled real dataset of extracellular electric traces from rat hippocampus. In conditions of relatively high difficulty (i.e., with additive noise and with similar action potential waveform shapes for distinct neurons) the method achieves significant improvements in clustering performance over a baseline waveform-only Gaussian mixture model (GMM) clustering on the semiartificial set (1.98% reduction in error rate) and outperforms both the GMM and a state-of-the-art method on the real dataset (5.04% reduction in false positive + false negative errors). Finally, an empirical study of two free parameters for our method is performed on the semiartificial dataset.

## 1. Introduction

Trace signals from extracellular electrodes implanted in a population of neurons are extremely valuable for studying the behavior of neurons and are used as the primary input source for motor and communication prostheses based on brain-computer interfaces [1, 2]. Given a trace of extracellular electric potential signals, neural spike sorting is the task of detecting neuronal action potential events in the form of “spikes” in the extracellular signal and identifying which neurons or neuronal clusters produced each spike in the trace. Many approaches to the spike sorting problem are based on discriminating between neuronal units by extracting features from spike waveforms and forming clusters in the feature space. This includes manual spike sorting approaches, where an expert visually identifies clusters of spike waveforms, and automatic approaches, which are typically based on rigorous signal processing and statistical modeling for clustering or classification. Many studies and practical implementations confirm the effectiveness of waveform-based spike sorting approaches; thorough reviews are given in [3, 4]. While approaches that use only the waveform shape have been successful, these methods are particularly susceptible to some very common sources of errors in intracortical BCIs. Distinct neurons having similar waveform shapes, changes in time-domain, and parameterized waveform shapes due to movement of the electrodes and excessive background noise all have a direct negative impact on the performance of waveform-only spike sorting methods.

An ensemble of neural spike trains, one for each neuron or neuronal cluster in the vicinity of the electrode, is typically the most important result of the spike sorting operation [5]. Estimates of the firing rates of neural spike trains, as well as stochastic point process models of the spike trains themselves, are used to make inferences about populations of neurons [6–8] and for decoding in intracortical BCIs [9–13]. While modeling and analysis of spike trains is usually associated with neural decoding, a number of recent studies have advocated incorporating neural spike trains (and other temporal information) into the process of spike sorting. A complete maximum-likelihood framework based on a variant of hidden Markov models (HMMs) is described in [14] to model neuronal bursting behavior. The sparse HMM framework proposed in [14] models counts of neural firing events in equally spaced time frames. In another study, extracellular traces were divided into short-duration time segments to model the nonstationarity of neuronal action potential waveform features [15]; Viterbi decoding was then used to find the optimal clustering across time segments. Several studies have explicitly incorporated models of interspike interval (ISI) durations into spike sorting [16, 17]. Stationary models of spike amplitudes and ISI durations are used in [16], while HMMs are incorporated in [17] to model the time-varying firing behavior of each neuron. Finally, a joint model of waveforms and firing rates, as modulated by a function of a known covariate quantity, was introduced in [18]. In [16, 17], Markov chain Monte Carlo distribution sampling was used for model inference.

In this paper, we detail a novel, probabilistic framework for neural spike sorting, based on stationary, stochastic ISI models of neural spike trains. A preliminary version of this work was presented previously in conference proceedings [19, 20]. Our method for spike sorting introduces a joint probabilistic model of parameterized spike waveforms and the occurrence times of an ensemble of neural spike trains. We derive a novel likelihood model and a procedure for clustering and parameter estimation and then evaluate the model on both fully labeled semiartificial neural data and partially labeled real data.

In Section 2 we describe our methods in detail. In Section 2.1, we define neural spike sorting as a latent-variable problem with three variables: the set of observed waveforms, the observed firing times, and hidden neuronal cluster labels. We give likelihood expressions for the three variables, and formulate the solution as maximizing their joint likelihood. We model the set of observed firing times as the aggregation of an ensemble of neural spike trains, one for each neuronal cluster, using a stationary, stochastic interspike interval model for each spike train. We then express the likelihood of the observed firing times as the joint likelihood of *K* neural spike trains. In Section 2.2, with the likelihood model defined, we derive a novel iterative method for clustering the data by finding the model parameters that maximize the joint likelihood of the firing times, waveforms, and hidden labels. For computational efficiency, the clustering method depends on iterating forward in time and keeping a short history of recent firing times at each stage.  Section 2.3 gives detail about our choices for the probability distributions used in the likelihood model, and Section 2.4 explains the meaning and use of the procedure's two hyperparameters.

Finally, in Section 3, we evaluate our method on publicly available datasets. We first use realistic, semiartificial data (i.e., real spike waveforms, with synthesized firing times), with a complete set of labels, to evaluate the performance of our spike sorting procedure versus a state-of-the-art method. We then evaluate our procedure on a completely real dataset, consisting of extracellular traces, and an intracellular trace for one neuron.

## 2. Methods

The task of neuronal action potential identification, or spike sorting, can be seen as a latent variable problem where the set of detected firing times and corresponding action potential waveforms are observed in an extracellular electric trace, and the identity of the underlying neurons is a hidden variable. In the remainder of this section, we describe a new approach to the spike sorting problem, where we model the set of observed, threshold-crossing neuronal firing times as the aggregation of multiple hidden point processes, one for each neuron. We use an iterative procedure to estimate the maximum likelihood sequence of states based on the set of observed action potential waveforms and firing times.

### 2.1. Likelihood Model

Let the vector **z** = {*z*_*i*_}_*i*=1_^*N*^ be the time occurrences of *N* observed, threshold-crossing events corresponding to firings of a population of *K* cortical neuronal clusters in the vicinity of the electrode. Let **X** = {**x**_*i*_}_*i*=1_^*N*^ be the set of corresponding parameterized action potential waveforms, where each vector **x**_*i*_ has dimension *D*, and let **c** be an *N*-length, discrete-valued vector containing the set of unknown neuronal labels corresponding to each observed event.

We define the posterior probability *P*(**c** | **X**, **z**) as follows:

(1) $$\begin{array}{c} P\left(c\;|\;X,z\right)=\frac{P\left(X,z,c\right)}{P\left(X,z\right)}\propto P\left(X,z,c\right), \end{array}$$

where we note that the term *P*(**X**, **z**) in (1) does not vary with respect to **c**.

The optimal sequence $\hat{c}$ thus satisfies

(2) $$\begin{array}{c} \hat{c}=\underset{c}{\arg\;\max}\;P\left(X,z,c\right). \end{array}$$

**Figure 1:**
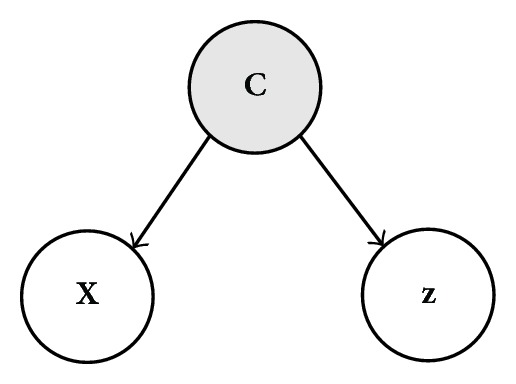
Statistical dependencies for parameterized waveforms **X**, occurrence times **z**, and labels **c**.

The graphical model in Figure 1 illustrates the assumptions about the statistical dependencies between the observed variables **X** and **z** and the latent variable **c** that we will use in our modeling framework. The figure illustrates that the observed variables in **X** and **z** depend on the hidden labels in **C**, but not on each other. On this basis, we express the likelihood *P*(**X**, **z**, **c**) as follows:

(3) P(X,z,c)=P(X,z ∣ c)P(c)

(4) =P(X ∣ c)P(z ∣ c)P(c),

where the terms *P*(**X** | **c**) and *P*(**z** | **c**) express the likelihood of the observed set of extracted neuronal waveforms and their corresponding occurrence times, respectively, given a sequence of neuronal labels **c**, and *P*(**c**) is the likelihood of the sequence itself.

In all experiments, we model the parameterized action potential waveform for each neuronal cluster as a single, multivariate Gaussian with parameters *θ* = {*μ*, Σ}, such that the waveform likelihood for cluster *j* is given by *p*(**x**; *θ*_*j*_) = *𝒩*(**x**; *μ*_*j*_, Σ_*j*_) and the likelihood for the complete set of waveforms is given by

(5) $$\begin{array}{c} p\left(X\;|\;c\right)=\overset{N}{\underset{i=1}{\prod }}p\left(x_{i};\theta_{c_{i}}\right). \end{array}$$

We characterize the temporal behavior of a population of *K* neuronal clusters by modeling the set of neuronal firing times **z** as the aggregation of *K* independent point processes (**t**_1_, **t**_2_,…, **t**_*K*_), where each **t**_*k*_ = {*z*_*j*_}_*j*∈*c*_*k*__ is the subset of **z** corresponding to firings of the *k*th neuronal cluster. It is convenient to model the likelihood *P*(**t**_*k*_) based on the distribution of interspike interval durations. Let *f*_*k*_(*τ*; *ϕ*_isi_) be a probability density function with parameter set *ϕ*_isi_, characterizing the distribution of the continuous, univariate time period *τ* = *t*_*k*,*i*_ − *t*_*k*,*i*−1_ between two consecutive firings of neuronal cluster *k* occurring at times *t*_*k*,*i*_ and *t*_*k*,*i*−1_. Assuming that interspike interval durations are independent and identically distributed, the likelihood *P*(**t**_*k*_) can be expressed as

(6) $$\begin{array}{c} P\left(t_{k}\right)=w_{k}\left(t_{k,1};\phi_{\text{init}}\right)\overset{N_{k}}{\underset{i=2}{\prod }}f_{k}\left(t_{k,i}-t_{k,i-1};\phi_{\text{isi}}\right)g_{k}\left(t_{k,N_{k}};\psi \right), \end{array}$$

where *N*_*k*_ is the number of neuronal firings in **t**_*k*_, *w*_*k*_(*t*; *ϕ*_init_) is the distribution of the first firing time *t*_*k*,1_, and *g*_*k*_(*t*_*k*,*N*_*k*__) = ∫_*T*_^*∞*^ *f*_*k*_(*x* − *t*_*k*,*N*_*k*__; *ϕ*_*k*_)*dx* is the distribution of the last firing time *t*_*k*,*N*_*k*__, where *T* is the total time length of the dataset [21]. We model the likelihood *P*(**z** | **c**) of the complete set of firing times in terms of the joint occurrence of all class-conditional firing times; that is, *P*(**z** | **c**) = *P*(**t**_1_, **t**_2_,…, **t**_*K*_). Since we have assumed that these *K* point processes are independent, we say

(7) $$\begin{array}{c} P\left(z\;|\;c\right)=P\left(t_{1},t_{2},\ldots ,t_{K}\right)=\overset{K}{\underset{k=1}{\prod }}p\left(t_{k}\right). \end{array}$$

The last term in (4), *P*(**c**), is the likelihood of the set of neuronal firing labels. It is important to note that since we have assumed that each neuronal cluster fires independently, all temporal modeling is expressed in terms of firing times and interspike intervals. Thus, unlike a hidden Markov model, we do not apply any explicit statistical modeling to the sequence of labels, and the likelihood *P*(**c**) is simply given by

(8) $$\begin{array}{c} P\left(c\right)=\overset{N}{\underset{i=1}{\prod }}P\left(c_{i}\right). \end{array}$$

### 2.2. Clustering and Parameter Estimation

**Figure 2:**
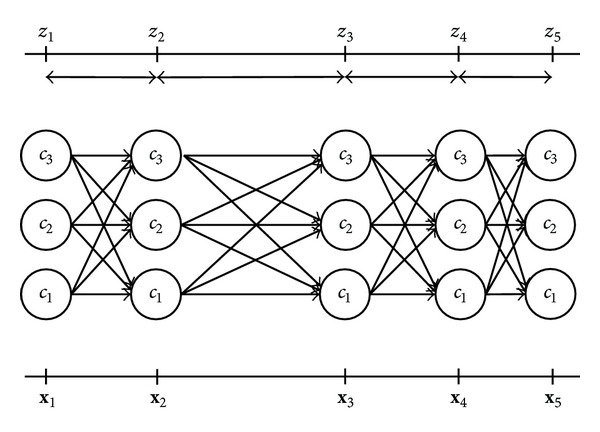
Lattice structure for clustering and parameter estimation.

We can represent the dynamic relationship between **X**, **c**, and **z** with the lattice structure depicted in Figure 2. Figure 2 depicts a dataset consisting of *K* = 3 neuronal clusters, with *N* = 5 observed firing times in **z** and corresponding action potential waveforms in **X**. The lattice structure is similar in appearance to the commonly used HMM trellis but has some important differences. Particularly, since we do not explicitly model transitions between states and all temporal modeling is based on *P*(**z** | **c**), uneven horizontal spacing is used to illustrate observed interarrival durations in **z**.

For the spike sorting task, we seek to exploit both the action potential waveform shape in **X** and the temporal information in **z**. To find the maximum likelihood sequence $\hat{c}$ as defined in (2) we use an approximate, iterative procedure to find the best path through the state space depicted in Figure 2. The procedure is initialized with a clustering based on the set of action potential waveforms only.

Given a set of parameters *λ* = {*θ*, *ϕ*_init_, *ϕ*_isi_}, we determine the maximum likelihood state sequence $\hat{c}$ by deriving a recursive expression for the joint likelihood *P*(**X**, **z**, **c**). Let the notation *P*({**x**_*i*_}_*i*=1_^*n*^, {*z*_*i*_}_*i*=1_^*n*^, {*c*_*i*_}_*i*=1_^*n*^) indicate the joint likelihood of the first *n* data points, such that the likelihood of the full set of *N* data points is given by *P*(**X**, **z**, **c**) = *P*({**x**_*i*_}_*i*=1_^*N*^, {*z*_*i*_}_*i*=1_^*N*^, {*c*_*i*_}_*i*=1_^*N*^). We decompose *P*({**x**_*i*_}_*i*=1_^*n*^, {*z*_*i*_}_*i*=1_^*n*^, {*c*_*i*_}_*i*=1_^*n*^) as follows:

(9) P({xi}i=1n,{zi}i=1n,{ci}i=1n)  =P(xn,zn,cn,{xi}i=1n−1,{zi}i=1n−1,{ci}i=1n−1)  =P(xn,zn,cn ∣ {xi}i=1n−1,{zi}i=1n−1,{ci}i=1n−1)   ·P({xi}i=1n−1,{zi}i=1n−1,{ci}i=1n−1)  =P(xn ∣ cn,{xi}i=1n−1,{zi}i=1n−1,{ci}i=1n−1)   ·P(zn ∣ cn,{xi}i=1n−1,{zi}i=1n−1,{ci}i=1n−1)   ·P(cn ∣ {xi}i=1n−1,{zi}i=1n−1,{ci}i=1n−1)   ·  P({xi}i=1n−1,{zi}i=1n−1,{ci}i=1n−1),

where we have used the statistical dependency assumptions given in (4) and (5) and illustrated in Figure 1. Assuming that action potential waveforms in **x**_*n*_ and hidden labels in *c*_*n*_ do not depend on any previous samples, we obtain

(10) P({xi}i=1n,{zi}i=1n,{ci}i=1n−1,cn=j)  =P(xn ∣ cn=j)·P(zn ∣ cn=j,ζj)·P(cn=j)   ·P({xi}i=1n−1,{zi}i=1n−1,{ci}i=1n−1).

Note in (10) that we have expressed the likelihood of an *n*-length label sequence ending in state *j* and that we have introduced a new variable *ζ*_*j*_. Given a label sequence ending in state *j*, the likelihood *P*(*z*_*n*_ | *c*_*n*_ = *j*, *ζ*_*j*_) depends on *ζ*_*j*_ < *z*_*n*_, which we define as the most recent, previous occurrence time of state *j*. To find *ζ*_*j*_, some bookkeeping is necessary. Specifically, at each iteration we retain the *L* highest likelihood label sequences or paths through the lattice structure illustrated in Figure 2 (the number of paths, *L*, is determined empirically). Each path contains only the most recent spikes occurring within a history time window, starting at time *z*_*n*_ − *τ*_win_ and ending at *z*_*n*_. The length *τ*_win_ of the history window is constant and is determined empirically. The likelihood in (10) is computed for the best *L* paths retained from the previous iteration. For a given path, the duration *z*_*n*_ − *ζ*_*j*_ is modeled with an interarrival distribution *f*_*j*_ as expressed in the second term of (6). If no previous occurrences of state *j* are found in a given path, we say that *ζ*_*j*_ = −*∞* and the distribution *w*_*j*_ (expressed in the first term in (6)) for the first firing time is used instead, with the window length *τ*_win_ as its argument. This is expressed in (11):

(11) p(zn ∣ cn=j,ζj)  ={wj(τwin  ϕinit),ζj=−∞fj(zn−ζj  ϕisi),otherwise.

*Iterative Procedure.* Though our spike sorting method uses both spike waveforms and firing times, we must initialize the procedure using spike waveforms only. We model the waveforms in **X** as a Gaussian mixture model (GMM) and find the maximum likelihood waveform parameters using the expectation-maximization (EM) algorithm to produce an initial clustering. Based on the initial clustering, we estimate parameters *ϕ*_isi,*j*_ for the interarrival distribution *f*_*j*_ and *ϕ*_init,*j*_ for the first-firing distribution *w*_*j*_, for each neuron *j*. For the first firing and interarrival distributions *w*_*j*_ and *f*_*j*_, we use the exponential and lognormal probability density functions, respectively. We then assign each data point **x**_*i*_ to the maximum a posteriori GMM component to produce a clustering and estimate parameters *λ* = {*θ*, *ϕ*_isi_, *ϕ*_init_} based on the clustering. The 3-step procedure is then as follows.

1. Decode with parameters *λ* and produce a segmentation.
2. Estimate parameters *λ*_new_ based on the segmentation.
3. Reiterate until convergence.

### 2.3. Probability Distributions

A breakdown of the probability distributions and their parameters used in all experiments is given in Table 1. We model parameterized action potential waveforms for each cluster as single, multivariate Gaussians and model interarrival durations with the conditional distribution expressed in (11). For *w*_*j*_, the distribution of the “first firing” after a long duration, we use a simple Poisson distribution *w*_*j*_(*k*; *βt*) = (*βt*)^*k*^ *e* ^−*βt*^/*k*!|_*k*=1_, with duration parameter *β* and event count *k* = 1. For the ISI distribution *f*_*j*_, we use a log-normal density with parameters *μ* and *σ* ^2^. The log-normal density has been shown to have a superior empirical fit to neuronal ISI durations having a necessary minimum refractory period [16, 17].

### 2.4. Parameters *L* and *τ*

In addition to the distribution parameters *λ* = [*θ*, *ϕ*_init_, *ϕ*_isi_], our procedure has two free parameters *L* and *τ*, which are determined empirically; these are the number of paths and the history window length, respectively. The number of paths *L* is typically chosen according to a trade-off of accuracy against speed and memory usage. We choose the window length *τ* such that the “*ζ*_*j*_ = −*∞*” condition in (11) occurs rarely. *τ* is chosen to be larger than an interarrival duration *t*_*k*,*i*_ − *t*_*k*,*i*−1_ for any neuron *k* with high probability. To estimate *τ* we fit a lognormal distribution to each neuronal cluster based on an initial waveform-only clustering of the data and choose *τ*_*k*_ to cover 99% of the area under the lognormal ISI curve for neuron *k*. The history window *τ* is then simply *τ* = max⁡_*k*_ *τ*_*k*_. The general expression for the log-normal density function for a variable *t* with parameters *μ* and *σ* ^2^ is given by

(12) $$\begin{array}{c} t\sim \text{Log}\;\text{Norm}\left(t;\mu ,\sigma^{2}\right)=\frac{1}{t\sigma \sqrt{2\pi }}\exp\left[-\frac{1}{2}{\left(\frac{\log t-\mu }{\sigma }\right)}^{2}\right]. \end{array}$$

Given a set of univariate, Gaussian-distributed data *x* ~ *𝒩*(*x*; *μ*, *σ* ^2^), if *χ* is the logarithm of *x* then, by definition, *χ* is log-normal distributed, such that *χ* = log⁡(*x*) ~ Log Norm(*χ*; *μ*, *σ* ^2^). The log-normal parameters *μ* and *σ* ^2^ are then the mean and variance of exp⁡(*χ*), respectively. The log-normal distribution is supported on the range [0, *∞*) and has been used successfully to model neuronal interspike interval durations [16, 17].

## 3. Experimental Results

Given a real, continuous extracellular trace, it is typically impracticable to obtain a complete set of ground truth labels since it cannot be directly observed which neuron caused each action potential spike in the trace. This makes evaluation for spike sorting difficult in most nontrivial cases. Synthetic extracellular traces, which are often partially composed of real data, provide fully labeled datasets useful for development and evaluation of spike sorting methods. When fully authentic data are desired, however, it is possible to collect data using both an extracellular electrode and a carefully placed intracellular electrode in one neuronal cell to obtain a partial ground truth labeling. Spikes on an intracellular electrode identify the firing times of one neuron with near certainty. In Sections 3.1 and 3.2, we apply our spike sorting method to two publicly available sets of cortical extracellular traces to demonstrate its performance. One of these datasets is real and partially labeled, and the other is semiartificial and fully labeled. Finally, in Section 3.3, we perform an empirical study of the parameters *L* and *τ* using the semiartificial, fully labeled dataset.

### 3.1. WaveClus Semiartificial Dataset

**Figure 3:**
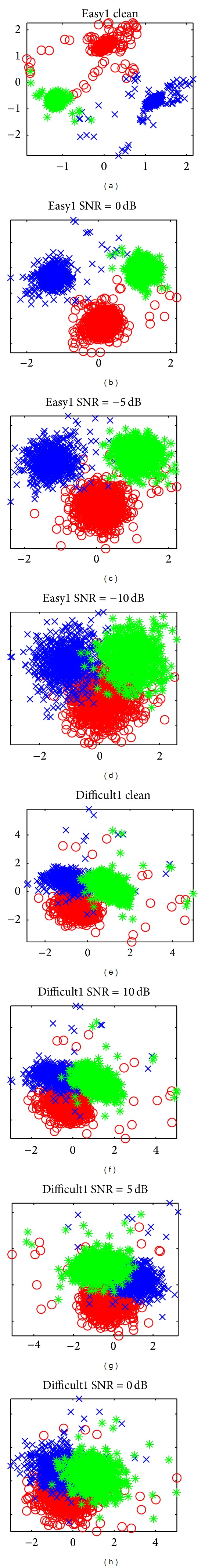
First 2 PCA coefficients of action potential wave forms plus noise at various SNR levels for the “Easy1” and “Difficult1” semi-artificial data sets.

We evaluate our spike sorting methods with labeled data collected, in part, from the publicly available WaveClus artificial dataset [22]. We use randomly selected action potential waveforms from “Example 1” and “Example 2” subsets, hereafter referred to as “Easy1” and “Difficult1,” respectively. Each data subset consists of *K* = 3 neuronal clusters with characteristic action potential waveform shapes drawn from a library of templates. The 3 characteristic waveforms in the “Difficult1” set are similar to each other in shape and are generally more difficult to separate than in the “Easy1” set. All of the WaveClus datasets contain realistic additive background noise at varying power levels. For our spike sorting experiments, we added additional Gaussian noise to the baseline data at various SNR levels. We use principal components analysis (PCA) for dimensionality reduction in all experiments. Scatter plots of the first 2 principal components are given in Figure 3 for both data subsets under various noise conditions.

All subsets of the WaveClus dataset contain 3 neuronal clusters with artificial firing times having identical firing rate statistics. For our experiments, we generated firing times according to a Monte Carlo sampling of 3 independent log-normal distributions, resulting in a dataset of 2483 firing times 24 seconds in length. A minimum 3-millisecond interval duration was enforced to model the refractory period for all clusters.

**Figure 4:**
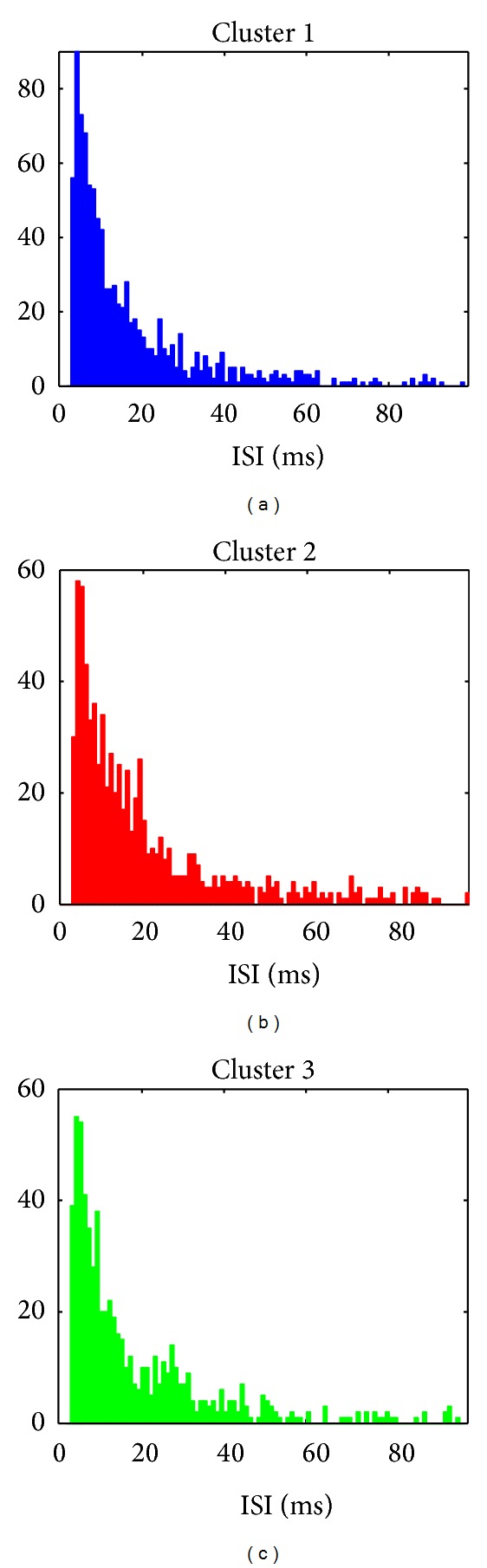
Inter-spike interval histograms for both the “Easy1” and “Difficult1” data sets. Spike firing times were generated according to a log-normal ISI distribution with parameters listed in Table 2, and a minimum refractory period of 3 msec.

Table 2 gives the simulation parameters, *μ* and *σ* ^2^, we used to generate interspike interval durations, along with the mean, in milliseconds, of the generated data. The parameters listed in Table 2 were determined by computing the sample mean and variance of putative log interarrival times taken from another publicly available dataset. (These data were collected in the Laboratory of Dario Ringach at UCLA and downloaded from the CRCNS website.) Plots of interspike interval histograms are given in Figure 4.

*Results.* To evaluate the accuracy of our method for spike sorting, we compute the classification accuracy of the best match between the set of true clusters and the set of putative clusters identified by our procedure. Quantitative performance results for the WaveClus dataset are given for the baseline GMM procedure and for our joint waveform and firing rate method in Table 3. We compare our proposed approach to a GMM baseline clustering (i.e., the waveform-only initialization) and to the state-of-the-art superparamagnetic clustering or “WaveClus” method [22]. Gaussian noise was added to both the “Easy1” and “Difficult1” datasets at various SNR levels with the original WaveClus dataset labeled “Clean” in the table.

Overall, we find that our method, which extends the waveform-only baseline by incorporating a hidden point process model for each neuron, reduces the error rate in the presence of noise. The error rate for the “Easy1” dataset at higher SNR levels (SNR > 0 dB) is not significantly changed from the baseline initialization by our joint waveform and firing rate method with respect to the initial clustering, which was already quite low (less than 1.0% error). The absolute difference in error rate for the baseline GMM clustering and our proposed method is less than 0.05% (i.e., 1 out of 2483 spikes in the dataset) at higher SNR levels. Since this dataset was particularly easy to classify, we added noise at −5 dB and −10 dB SNR. In the presence of high noise (−10 dB SNR), we reduce the error rate from 8.18% to 6.20% by incorporating temporal information.

A similar trend is seen with the “Difficult1” dataset, but at higher SNR levels. At 10 dB SNR and 5 dB SNR, we reduce the error rate with respect to the baseline by 1.37% and 1.57%, respectively. However, when SNR is reduced to 0 dB for the “Difficult1” dataset, we see a significant increase in the error rate. The WaveClus method, however, performs significantly better on this dataset overall.

### 3.2. Continuous Extracellular Traces

**Figure 5:**
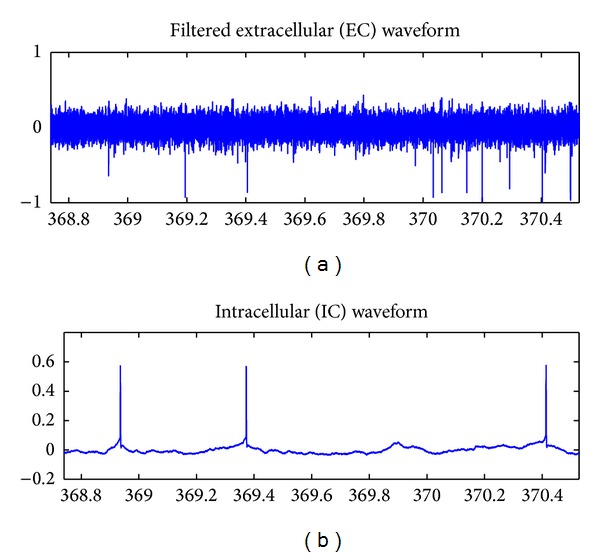
Simultaneous extracellular (EC) and intracellular (IC) electric potential traces taken from Dataset 1 of completely real data set HC1. True firing times of the “IC neuron” are known with near certainty.

To evaluate our methods on real, continuous data, we use a publicly available dataset of cortical electrical traces taken from hippocampus of anesthetized rats, hereafter referred to as “HC1” [23]. The HC1 dataset consists of traces of extracellular (EC) electric potentials, as well as intracellular (IC) traces for 1 of *K* neurons in the vicinity of the EC electrodes. We use two subsets of the HC1 dataset, each 4 minutes in length, to evaluate our spike sorting procedure. Both the EC and IC electric potential signals for Datasets 1 and 2 were recorded at a sample rate of 20 kHz. We use a high-pass filter to eliminate waveform drift for the extracellular signals. A plot of a 1.79 s segment of simultaneously recorded EC and IC signals from Dataset 1 is given in Figure 5. Three peaks in the lower panel of Figure 5 indicate firing times of the “IC neuron” and correspond to 3 of the peaks in the EC signal in the upper panel.

*Methodology.* We detect neuronal action potentials as “spikes” in the extracellular signal exceeding a threshold of 5*σ*, where *σ* is an estimate of the standard deviation as defined in [22]. In all spike sorting experiments, we extract observed action potential events as 4 ms waveforms centered at the peak point in each extracellular spike. To locate spikes on the intracellular channel in each data subset, we take the first backward difference of the IC signal and apply a peak-picking algorithm to it. EC action potentials occurring within 1 ms of an IC spike are labeled as belonging to the IC neuron. In Dataset 1, we detected 1090 total extracellular firings and 396 intracellular firings. In Dataset 2, we detect 3017 EC firings and 1100 IC firings. In each dataset, there are *K* = 3 neuronal clusters.

**Figure 6:**
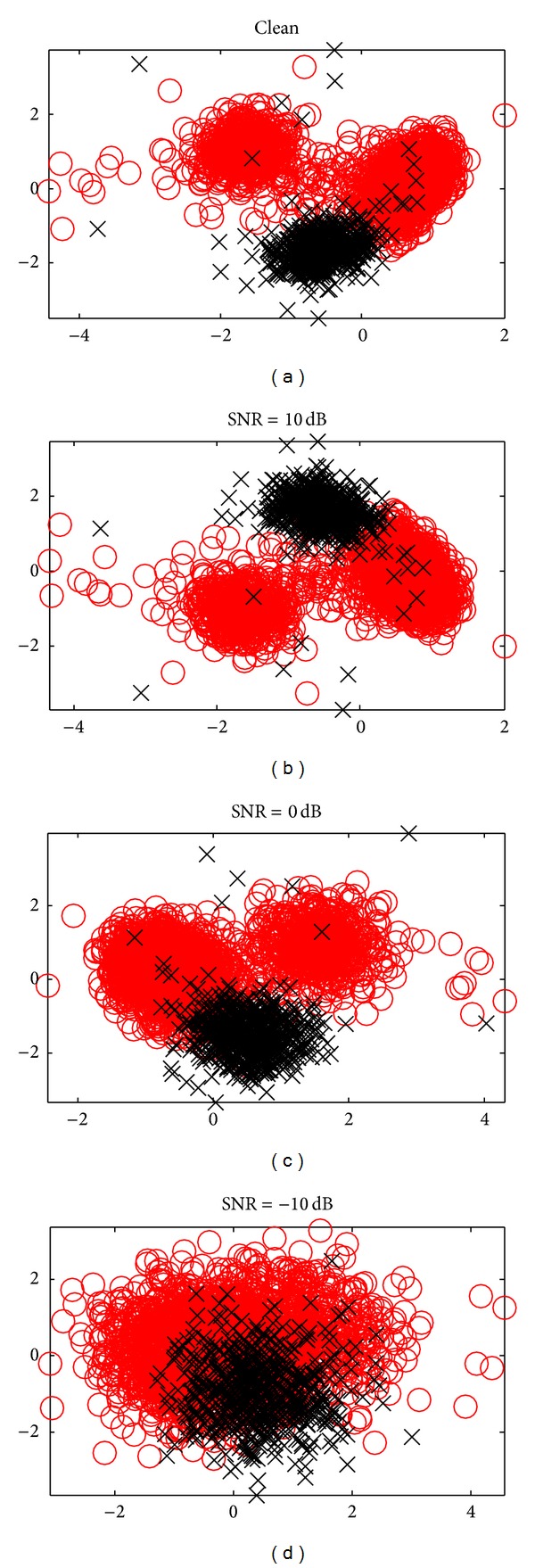
PCA waveform features plus noise for Dataset 2 of HC1. Features for the “IC neuron” are shown with black “X” markers.

For EC waveforms, we use principal components analysis (PCA) for dimensionality reduction. We keep the first 3 principal components as features for **X**, the matrix of observed action potential waveforms. For our spike sorting experiments, we add Gaussian noise to the waveform at various SNR levels before applying PCA. Scatter plots of the first two principal components at various SNR levels are given in Figure 6; extracellular waveform features corresponding to firings of the IC neuron are distinguished with black “X” markers. 

*Evaluation.* Given only a partial labeling of the data, we can evaluate the performance of a spike sorting result in terms of false positive (FP) and false negative (FN) errors for the labeled IC neuron. When a spike corresponding to the IC neuron is misclassified, a FN error is counted; inversely, when a spike is erroneously classified as belonging to the IC neuron, a FP error is counted. The error rate is defined as the sum of the FN and FP counts, divided by the number of EC firings. 

*Results.* Quantitative performance results, in terms of the total (FP + FN) error rate, are given in Table 4. For the WaveClus method, we use both a wavelet-based parameterization (the default choice for the WaveClus software package) of action potential waveforms and PCA features for a more direct comparison with the other results.

Our proposed joint waveform and firing rate approach performs the best in all cases except at the lowest SNR level for both data sets 1 and 2. The WaveClus method results in very high FN error counts, but low FP counts (only the total error rate, FP + FN, is shown in Table 4). We see a larger improvement over the GMM baseline (nearly 5% at some levels) for Dataset 1, which is ostensibly the more difficult set of the two, as evidenced by higher overall error rates for all classifiers.

### 3.3. Empirical Study of Parameters

Our clustering approach involves retaining a large number of paths, *L*, and a time history window of length *τ* of recent firings. In all of our previously reported results, we have used a fixed value of *L* = 10000 paths chosen on the basis of computational and memory constraints. The value of *τ* was determined to cover 99% of the area of the estimated interspike interval probability density curve, as described in Section 2.4. In this section, we perform an empirical study on the impact of our two free parameters *L* and *τ* on spike sorting performance on the WaveClus dataset. We first study the impact of increasing *L* on classification accuracy with *τ* determined as described in Section 2.4. Then, with a fixed value of *L* (we chose *L* = 1000) we study the effect of *τ* on the accuracy over a reasonable range. To evaluate, we simply compute the classification accuracy for the best match between the set of true clusters and the putative result.

**Figure 7:**
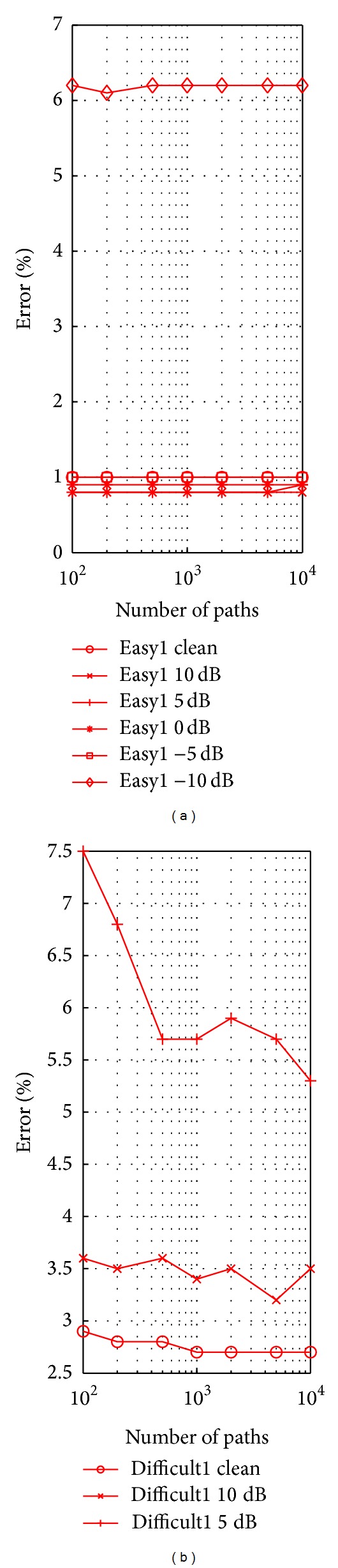
Error rate versus *L*, the number of paths.

In Figure 7, we plot the classification error rate for our spike sorting method with the value of *τ* determined empirically for values of *L* ranging from 100 to 10000 paths on a logarithmically scaled ordinate axis. For the Easy1 dataset and at higher SNR levels, the performance is largely unaffected by the number of paths *L*. For Difficult1 dataset, the value of *L* has a much more significant impact on the outcome. The impact is more pronounced for lower SNR levels, reducing the error rate for Difficult1, 5 dB SNR case, from 7.5% to 5.3% across the extremes of the range. The results in Figure 7 illustrate a trade-off of accuracy against computation and memory requirements, both of which increase with *L*, and suggest that except in difficult, high-noise conditions, the number of paths *L* can be effectively reduced with minimal impact on performance.

**Figure 8:**
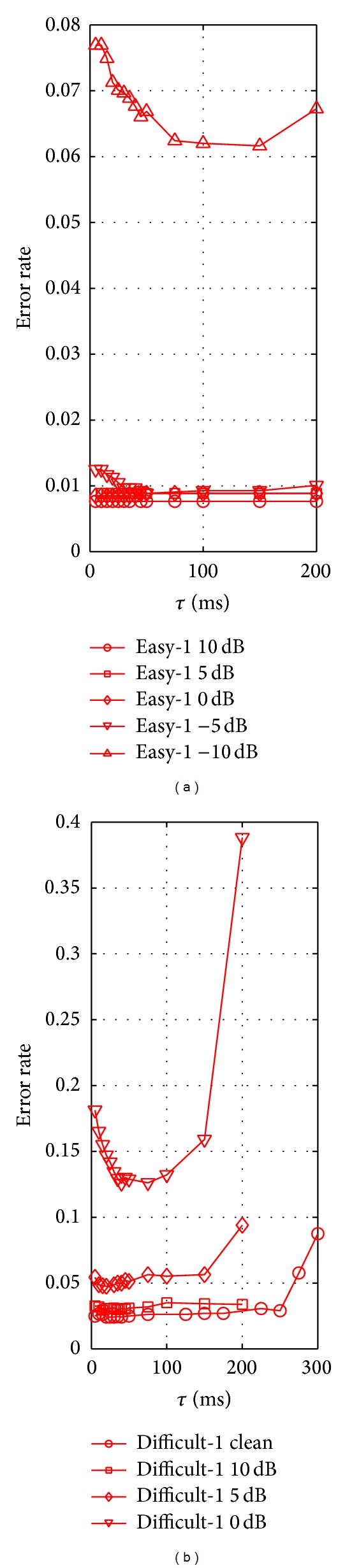
Error rate versus the window length *τ*.

To evaluate the impact of the history window length *τ*, we apply our spike sorting procedure to the WaveClus data over a range of values, this time holding the number of paths *L* fixed. Choosing a value of *L* = 1000, we implement our procedure for values of *τ* ranging up to 300 ms. Plots of the error rates obtained on the WaveClus dataset are given in Figure 8. As with *L*, the accuracy is less sensitive to the value of *τ* in easier, low noise conditions.

## 4. Discussion

Our probabilistic method for spike sorting is motivated by the idea that both the spike waveforms and their corresponding firing times constitute observed data useful for making inferences about the underlying hidden process of which neurons produced them. In doing so, we incorporate relevant data largely unused by many traditional spike sorting approaches. We combine a single Gaussian model of spike waveforms and a first-order renewal process model of firing times for each neuron into a joint probabilistic model of several neurons in the vicinity of an electrode. The observed firing times are modeled as the aggregation of *K* independent point processes.

Our method is designed to improve accuracy over waveform-only spike sorting methods, especially under conditions to which these methods are particularly sensitive, such as the presence of high noise and having similar wave shapes for distinct neurons. Our method consists of a joint probabilistic model of multivariate single Gaussians for the first two principal components of neural spike waveforms and log-normal and Poisson ISI models for the neural spike trains. To study the impact of incorporating neural spike train models we compared the performance of our method to a waveform-only clustering of multivariate Gaussians using the expectation-maximization GMM (EM-GMM) algorithm and the state-of-the-art WaveClus algorithm. First, we evaluated all methods on a modified version of the semiartificial WaveClus dataset, for which all neuronal labels and firing times are known with certainty. While the state-of-the-art WaveClus method outperforms our method on this dataset, we achieved significant reductions in the error rate versus the EM-GMM waveform-only model in two general cases: (1) “Easy” waveforms (i.e., distinct wave shapes for distinct neurons) in the presence of high additive background noise and (2) “Difficult” waveforms with relatively low noise. In both of these cases, there is significant overlap in the waveform feature space, but the firing times, which are determined through detecting threshold-crossing spikes, are not significantly impacted. Under these conditions, our model uses all of the information and converges on a better result after an iterative procedure.

In the second dataset, composed of completely real data, our method consistently achieves better performance than both the baseline waveform-only EM method and the WaveClus method. It should be restated here that error rates reported on this dataset reflect only one out of three neurons and are only a partial measure of total classification or clustering performance. We achieve considerable reductions in the error rate versus the baseline on Dataset 1 at all noise levels and on Dataset 2 in relatively high noise (i.e., −5 dB SNR). As with the “Easy” data set from the semiartificial WaveClus data, when the baseline EM-GMM method already achieves a low error rate, our proposed method achieves only a small improvement. With Dataset 1 and the high noise or “Difficult” configurations of the WaveClus set, the baseline error is already relatively high, and our model reduces it significantly (as much as 1.98% for the WaveClus data and 5.04% for Dataset 1). However, in extremely high noise (−10 dB for both real datasets and 0 dB for the “Difficult” WaveClus dataset) our method obtains high error rates (greater than 30% error rate). The likely cause for the failure in extremely high noise is that our method is sensitive to its initial clustering (recall that the waveform-only EM-GMM method is actually used to provide the initial clustering for our proposed procedure) and our iterative parameter estimation procedure described in Section 2.2 is unable to converge. Therefore, our spike sorting procedure, which uses models of neural spike trains, achieves the most impactful reductions in error rate on problems of intermediate difficulty.

Contributions of this work include modeling the likelihood of the observed point process of firing times as the joint likelihood of an ensemble of spike trains; a mathematical derivation of the joint likelihood of waveforms, firing times, and labels in terms of previous data points; and the derivation of an iterative clustering and parameter estimation procedure using a piecewise ISI likelihood expression. Our procedure for clustering and parameter estimation operates by alternately maximizing the data likelihood and estimating new parameters based on the result. While the basic idea is similar to expectation-maximization or Viterbi-based parameter estimation in HMMs, our procedure is suboptimal with respect to the data likelihood. Since our approach depends on retaining a large number of the highest likelihood paths, performance, then, depends on the available computational resources. For this reason, we studied the impact of the number of stored paths on spike sorting performance and found that, except in the most difficult, high-noise conditions, we could reduce the number of paths by a factor of 10 without a significant loss in accuracy.

It should be noted that some issues important to the spike sorting problem, and to clustering problems in general, have not been directly addressed here. Using datasets each containing the same number of neurons, we have not addressed determining the number of neurons automatically. Since we initialize our procedure with a waveform-only GMM clustering, determining the number of clusters using the Aikake information criterion (AIC), the Bayesian information criterion (BIC), or several other methods requires only a simple extension. However, the iterative clustering procedure can potentially prune out clusters dynamically. A measured approach to pruning out clusters (including initializing with a high number of clusters) is a topic for future study. Overlapping spikes, changes in waveform shape due to electrode drift, and waveform attenuation, especially due to bursting activity, are also important topics for future research. Though our findings suggest that our algorithm, which incorporates firing information, might be particularly robust to electrode drift and waveform attenuation, since it improves over the baseline with difficult waveform shapes and high noise, this should be verified experimentally in future research.

### 4.1. Comparisons to Related Work

In this paper, we have proposed and demonstrated a novel spike sorting method motivated by the idea that both observed spike waveforms and observed information about the timing of spike events are potentially useful for the spike sorting task. A number of other recent studies have introduced spike sorting methods based on this general idea and, in this section, we contrast these approaches and their merits to ours.

In [14], Sahani introduced a statistical model of neuronal action potentials, particularly well suited for “bursts” of neural firings, that is, sequences of very rapidly occurring action potentials. The method for spike sorting proposed in [14], termed Sparse Hidden Markov Models (SHMMs), is a special case of the well-known hidden Markov model (HMM), but with the restriction that the majority of outputs are expected to be in a null state corresponding to nonfirings. The SHMM, as applied to modeling neural firings in [14] involves partitioning the neural firing activity into relatively long (0.5 ms) equal length time bins. Transitions between states are then restricted in the HMM transition matrix in such a way as to model neural bursting behavior. The SHMM has multiple nonnull states to model changes in the amplitude expected during bursting behavior and multiple null states which effectively keep track of how much time has passed since the last firing. In this study, the author recommends using simpler models for nonbursting neural cells. Sahani's approach is different from ours in several important ways. Instead of explicitly modeling transitions between states using a discrete set of transition probabilities as with an HMM, we incorporate temporal information using a likelihood model of the continuous-valued interval between firings. This means that we model the precise timing of firing events, rather than grouping them into short time bins. Furthermore, our approach models the joint likelihood of an ensemble of point processes, which differs significantly from both the SHMM approach used in [14] and the general HMM approach as well.

Bar-Hillel et al. introduced a nonstationary method for spike sorting designed to account for the apparent nonstationary nature of spike waveform data [15]. The method, referred to in the study as a “chain of Gaussian mixtures,” first segments neural firing data into short frames, which are either equal in length or contain an equal number of neural firings. The authors fit a separate GMM to the data in each frame (thus assuming stationarity within the frame), compute a set of transition probabilities between frames, and finally solve for the best maximum a posteriori clustering across the whole dataset using a variant of the Viterbi algorithm. This study directly incorporates temporal information into the spike sorting operation and is particularly well suited to account for waveform drift, where the amplitude and shape of the spike waveform are affected by movements in the electrode over time. While this method incorporates temporal information into spike sorting, it does so not by modeling either time occurrences of neural firings or the neural firing rate. Rather, the method is essentially a nonstationary model of spike waveforms, in which waveform parameters change over time. Similar to HMMs, the approach in [15] explicitly models transitions between time frames, which is very different from our approach. Furthermore, although our approach is explicitly designed to model temporal information in the extracellular signal, it is actually a* stationary* probabilistic model. As discussed in Section 2.3, all of the parameters *λ* = {*θ*, *ϕ*_isi_, *ϕ*_init_} for the joint model belong individually to stationary distributions. The ISI and “first firing” distributions, which account for temporal information in the data, are both stationary models of point processes.

Ventura introduced a method in [18], in which neural firing rates are incorporated into the spike sorting operation. The method consists of a probabilistic model of spike waveforms and an analytic model *λ*_*i*_(*c*) of the firing rate for neuron *i* as modulated by covariate information in the value *c*, such as an applied stimulus or a tuning curve or some other known experimental condition that impacts neural firing rates. The firing rate model *λ*_*i*_(*c*) in Ventura's method, which can be either parametric or nonparametric, is used to determine the probability of each possible combination of the *K* neurons in the vicinity of the electrode firing in short, equal length time bins. Modeling all possible combinations of spikes makes the method particularly well suited to accounting for overlapping spike waveforms. Since firing activity is incorporated in the form of *λ*_*i*_(*c*), given in units of spike per msec, the method implicitly models neural firing as an inhomogeneous Poisson process. The paper introduces an expectation-maximization procedure for estimating all of the parameters of the model. In experiments with simulated data, Ventura showed that a parametric model of firing rates *λ*_*i*_(*c*) could be used when the form of the firing with respect to the covariate quantity is known and that a nonparametric model can be used in more general cases. Ventura's approach is based on simultaneously performing spike sorting and tuning in an integrated procedure and, as such, it depends directly on modeling some known experimental condition or covariate quantity; this is in contrast with our proposed approach which does not depend on modeling or observing any covariate information. Instead, we assume only a general analytic form of the observed firing times as point processes. Also, an important part of our approach is explicitly modeling the distribution of the observed firing times. We used the lognormal distribution in this paper for its facility in modeling the necessary minimum refractory period, but our method is not limited to this or any other distribution assumptions.

A probabilistic model of spike peak amplitudes and firings was presented by Pouzat et al. in [16]. In that study, the authors advocate modeling the temporal behavior of spike timings using a probability distribution for the interspike interval duration. Also, since it is known that spike amplitude depends on the elapsed time since the last firing, the paper models variation in spike peak amplitude based on ISI durations as well. Much of the development in this study focuses on the use of Markov chain Monte Carlo (MCMC) methods, which allow considerable flexibility in the choice of probability distributions for the data. The MCMC method consists of simulating the posterior density of the model parameters and sampling from that distribution for parameter estimation. The authors use a lognormal distribution for neural firing ISIs and evaluate their model on simulated data. In another study [17], the authors extend the MCMC methodology for spike sorting, this time using an HMM. In [17],* sequences* of ISI duration observations for each neuron were modeled with a 3-state HMM having the lognormal distribution as the emission probability density function for each state. The HMM method was applied to a real dataset, exhibiting bursty neural firings recorded from Purkinje cells in rats, achieving high accuracy. While these two studies use continuous-valued ISI distributions for temporal modeling, they use MCMC sampling for parameter estimation, in lieu of an analytical development for the model. While the MCMC methodology generally allows considerable freedom in constructing compound probabilistic models, methods developed analytically are inherently simpler and more transparent. A key contribution of our approach is the mathematical development of a recursive likelihood model of the data, including a piecewise ISI term, and an iterative procedure for clustering and parameter estimation based on that model.

### 4.2. Summary

We have developed a model of observed, threshold-crossing neuronal firing times as the aggregation of *K* point processes and incorporated it into a joint waveform- and ISI-based framework. We developed an analytic expression of the joint likelihood of the observed and hidden data and formulated a recursive expression for the likelihood at any time *n* in terms of previous data. The ISI likelihood at time *n* is a piecewise expression that depends on whether previous spikes occur in a time history window. We developed an iterative procedure for clustering the data and estimating parameters based on finding the best path through a lattice structure. Our method outperformed the baseline, waveform-only GMM in noisy and otherwise difficult signal conditions on a semiartificial data. On a completely real dataset, our proposed approach outperformed both the baseline and a state-of-the-art method. We showed that we can obtain improvements in accuracy and computational efficiency by tuning our model's 2 hyperparameters. Our future work includes developing more sophisticated methods of pruning the search space for the best path and developing a more rigorous clustering method.

## Figures and Tables

**Table 1:** Breakdown of parameter set *λ* = [*θ*, *ϕ*_init_, *ϕ*_isi_] and probability distributions for joint waveform and firing rate spike sorting.

Waveform	Gaussian	*θ*_*j*_ = {***μ***_*j*_, Σ_*j*_}
First firing [*w*_*j*_]	Poisson	*ϕ*_init,*j*_ = {*β*_*j*_}
ISI [*f*_*j*_]	Log-normal	*ϕ*_isi,*j*_ = {*μ*_*j*_, *σ*_*j*_^2^}

**Table 2:** Simulation parameters for interspike interval data.

Cluster	Parameters		Mean ISI (ms)
	*μ*	*σ* ^2^	
1	1.5814	2.4203	24.5342
2	2.1610	1.9380	30.0564
3	1.9651	2.7068	33.9112

**Table 3:** Classification error rates for the WaveClus semiartificial dataset.

Dataset	SNR	GMM	WaveClus method	Proposed
Easy1	Clean	0.93%	0.00%	0.97%
	10 dB	0.72%	0.00%	0.77%
	5 dB	0.89%	0.00%	0.85%
	0 dB	0.81%	0.21%	0.85%
	−5 dB	1.25%	0.52%	0.97%
	−10 dB	8.18%	3.47%	6.20%

Difficult1	Clean	3.18%	0.45%	2.58%
	10 dB	4.83%	0.94%	3.46%
	5 dB	6.89%	1.36%	5.32%
	0 dB	19.77%	20.0%	37.21%

**Table 4:** Classification error rates (FP + FN) for the HC1 dataset.

Dataset	SNR	GMM	WaveClus Method		Proposed
			Wavelets	PCA	
Dataset 1	Clean	10.64%	32.02%	17.98%	5.60%
	15 dB	9.73%	24.5%	16.79%	4.77%
	10 dB	9.73%	31.84%	21.47%	4.95%
	5 dB	11.28%	31.93%	32.02%	6.51%
	0 dB	10.19%	32.11%	31.56%	9.27%
	−5 dB	20.09%	32.11%	32.02%	15.23%
	−10 dB	31.93%	67.98%	31.93%	32.11%

Dataset 2	Clean	2.09%	8.49%	6.56%	1.86%
	15 dB	1.96%	7.56%	6.20%	1.76%
	10 dB	1.99%	7.59%	6.27%	1.89%
	5 dB	2.29%	19.62%	11.04%	2.06%
	0 dB	3.51%	19.42%	14.29%	3.45%
	−5 dB	6.99%	19.65%	19.52%	5.87%
	−10 dB	32.55%	19.59%	19.56%	30.53%
